# Ethics in Medicines: Exposing Unethical Practices and Corruption in All Sectors of Medicines Is Essential for Improving Global Public Health and Saving Patients’ Lives

**DOI:** 10.3390/medicines8090054

**Published:** 2021-09-14

**Authors:** George J. Kontoghiorghes

**Affiliations:** Postgraduate Research Institute of Science, Technology, Environment and Medicine, Limassol 3021, Cyprus; kontoghiorghes.g.j@pri.ac.cy

**Keywords:** ethics in medicines, global health care, patient care, corruption in medicines at all levels, tackling corruption, one world/one health, plagiarism, victimization, orphan diseases, orphan drugs, human rights

While great strides have been made in science and medicine ensuring better living standards and health care for most human beings, many issues still remain, which are responsible for an increase in mortality and morbidity rates of millions of people worldwide, despite that in most cases the causes are preventable. Some of these preventable causes include communicable, non-communicable, tropical and other diseases, hunger/malnutrition, environmental pollution, wars, domestic violence, suicides, motor accidents etc. [1]. These problems and their effects on health care can often result from human activities, national policies and other factors, such as for example maintenance of the great inequality amongst individuals and nations with about 10% of the world population owning 90% of the world wealth and resources [2]. Efforts by various organizations and countries to publicize and tackle these worldwide issues are gradually changing world public opinion, e.g., on the toxic effects on smoking, gas emissions and climate change, environmental pollution etc. In particular, in the era of the COVID-19 pandemic where national barriers proved ineffective for containing the transmission of the disease, it has once again been demonstrated the interdependence of nations on health issues, as well as the need for implementing worldwide policies on health and also the need for the construction of one world/one health model on health care and medicines [3,4].

The lack of access to inexpensive, effective, non-toxic medicines particularly for patients in poorer countries is a major contributor to the high rates of mortality and morbidity of millions of patients worldwide [4]. This is clearly demonstrated nowadays for example by the inequality between poor and rich nations in the access and supply to vaccines against the severe acute respiratory syndrome coronavirus 2 (SARS-CoV-2) and the absence of effective medications for treating many orphan and tropical diseases, as well as other diseases [1,2,5,6,7].

However, in addition to inequality in the access to medicines between nations there are many other hurdles to achieving sustainable global health policies for all human beings. One such major hurdle is corruption, namely the abuse of power by individuals/companies/organizations/institutions etc. for private gain. Corruption, dishonesty and fraud are unethical but are embedded in all national and global health systems causing drainage of public health funds and an increase in the rate of mortality and morbidity of patients [8]. Corruption is observed at all levels of public health and almost all associated sectors including governments, academia and health professionals, mass media, patient organizations etc. [4,8,9]. The level and scale of corruption is usually proportional to the level of the finance involved and is more pronounced in relation to the areas of medicines and medical devices, where annual sales exceed trillions of euros [4,9]. Similarly, many of the methods employed for financial gain are unethical but in many cases lie within the grey areas of the law and on the boundaries of legal/illegal activity [9]. These illegal activities can range from simple law violations to industrial espionage related to medicines and medical devices by national secret services against other countries in the name of “national interest”.

Implementation of universal health coverage worldwide cannot be achieved unless corruption is openly discussed and tackled at all levels. In particular, the participation and role of leading international organizations or groups such as the World Health Organization, the United Nations Development Program, the World Trade Organization, the World Bank, G7 group of counties etc. is essential in formulating policies, issuing directives and tackling corruption at all levels [8,10,11,12]. Such initiatives are not unprecedented. Similar steps have been taken through recent legislation on anti-corruption, transparency and accountability policies by national governments in the EU, USA and other countries, as well as investigating individuals and banks for “money laundering” and reducing waste, e.g., pollution from plastic products. In this context, similar legislation tackling corruption and safeguarding public health are more pressing for saving the lives of people than solving “money laundering” issues.

In relation to medicines and medical devices involving public hospitals/health centers, universities and related institutions, as well as other associated organizations, corruption involving private companies can be expressed in a number of different “lobbying procedures and ways” which include donations, bribes and kickbacks, embezzlement, fraud, plagiarism, political influence/nepotism, research grants, travel grants, conferences, medical writers, informal payments etc. [4,8,9,10,11,12,13,14,15,16,17,18]. In most of these unethical cases this mode of behavior is not clearly outlawed or challenged in courts by state authorities [9,13,14,15,16,17,18]. In contrast, most of the people involved in these activities consider such cases legitimate and in general state authorities turn a blind eye to them [9,13,14,15,16,17,18,19,20,21].

There are many cases in the medical and other literature, as well as the mass media where unethical and corrupt practices related to medicines and medical devices by public and associated organizations have been reported. The range of examples includes but is not limited to governments, international health organizations, regulatory authorities, patients organizations, elite medical journals, hospitals, universities, physicians and other public employees etc. [4,7,8,9,13,14,15,16,17,18,19,20,21,22,23,24].

It is the role and responsibility of academic and research communities in all countries to address corruption and highlight its implications in everyday life and in all levels, prioritizing the availability of health care and medicines for all. Most importantly it is the responsibility of the academic and research communities to safeguard public health and not only to identify and highlight such problems but also to employ the appropriate tools in academic research to list the criteria and find solutions against unethical and corrupt practices in all the sectors of public health including medicines. Within this context topics such as ethics, corruption and related issues should be taught in schools and universities and sophisticated research tools should be employed by research organizations, which should be similar or even better than the sophisticated, influential, powerful marketing methods used by mainly multinational companies for maximizing profits in medicines and public health care in general [4,7,9]. Furthermore, every effort should be made for instituting in addition to ethical committees, anticorruption units in public hospitals and universities, as well as the establishment of protective measures against fraud and corruption, transparency and accessibility and also for protecting against any form of victimization at all levels of persons or organizations exposing any form of unethical and corrupt practices [15,16,17,22,23,24].

In addition to corruption there are many other specialized and specific areas which prevent universal access to public health care and medicines or medical devices, such as for example the monopoly on patents for drugs, which in general allows rich countries to charge extravagant prices for new drugs thus limiting access to new drugs for patients in poor countries and patients with low income in most other countries [4,9,25,26,27]. Similarly, laws should be implemented or established to regulate and restrict the marketing and promotion of new patented medicines and medical devices, as it has been shown that not only is the expenditure on marketing a main reason for charging high prices by the pharmaceutical and associated industries but also a major source of corruption and unethical practices at all levels in public health care, academia and associated sectors [4,9].

It is hoped that eventually access to public health care and medicines, as well as food and water should be considered, recognized and treated worldwide as a human right similar to the other human rights recognized by the United Nations. With the above in mind and by aiming to achieve sustainable development goals in health, it is important to focus activities on the identification, disclosure and tackling methods as well as provide constructive suggestions for solving any unethical and corrupt practices of any form and at any level in academia, research and public health which are related to health care, medicines and medical devices. Overall, such activities could help to improve society, the general level of health of people and at the same time reduce morbidity and mortality worldwide.

## Data Availability

Not applicable.
